# Autonomous Internet of Things (IoT) Data Reduction Based on Adaptive Threshold

**DOI:** 10.3390/s23239427

**Published:** 2023-11-26

**Authors:** Handuo Zhang, Jun Na, Bin Zhang

**Affiliations:** 1 School of Computer Science and Engineering, Northeastern University, Shenyang 110167, China; 1910630@stu.neu.edu.cn; 2 Software College, Northeastern University, Shenyang 110167, China; najun@mail.neu.edu.cn

**Keywords:** data reduction, Internet of Things, Kalman filtering, concept drift detection

## Abstract

With the development of intelligent IoT applications, vast amounts of data are generated by various volume sensors. These sensor data need to be reduced at the sensor and then reconstructed later to save bandwidth and energy. As the reduced data increase, the reconstructed data become less accurate. Usually, the trade-off between reduction rate and reconstruction accuracy is controlled by the reduction threshold, which is calculated by experiments based on historical data. Considering the dynamic nature of IoT, a fixed threshold cannot balance the reduction rate with the reconstruction accuracy adaptively. Aiming to dynamically balance the reduction rate with the reconstruction accuracy, an autonomous IoT data reduction method based on an adaptive threshold is proposed. During data reduction, concept drift detection is performed to capture IoT dynamic changes and trigger threshold adjustment. During data reconstruction, a data trend is added to improve reconstruction accuracy. The effectiveness of the proposed method is demonstrated by comparing the proposed method with the basic Kalman filtering algorithm, LMS algorithm, and PIP algorithm on stationary and nonstationary datasets. Compared with not applying the adaptive threshold, on average, there is an 11.7% improvement in accuracy for the same reduction rate or a 17.3% improvement in reduction rate for the same accuracy.

## 1. Introduction

The rapid advancement in mobile smart hardware has enabled the creation of intelligent IoT applications, which generate a vast amount of sensor data [1]. The amount and geographic distribution of these data make them distinctive. Processing such data requires sending the data to a remote processor, such as a sink node, an edge device, or a cloud center, as sensor devices frequently lack the computing and storage power to do so [2]. These sensor data are often reduced at the sensor and then reconstructed at the data processor to save bandwidth and communication costs.

Research on data collection and reduction in wireless sensor networks (WSNs) aims to reduce IoT nodes’ energy consumption by reducing data transmission volume [3]. Data compression, data prediction, and data aggregation are the three main types of data reduction algorithms [4]. Data prediction is a more popular and effective strategy because it may achieve a substantial data reduction ratio in contrast with other strategies [5]. Data prediction executes by building a data prediction model with the same parameters at both the sensor and the remote processor. The data predicted by the sensor and the remote processor are identical at one time. Therefore, the sensor only needs to determine if the predicted data are accurate or not before uploading the data. No data transmission is necessary if the difference between the predicted value and the collected value is smaller than the reduction threshold. If not, the remote processor receives the data sensor gathered, and the prediction model is updated [4]. As the reduced data increase, the reconstructed data become less accurate. Usually, the trade-off between reduction rate and reconstruction accuracy is controlled by the reduction threshold, which is calculated by experiments based on historical data.

Considering the dynamic nature of IoT, a fixed threshold can rarely maintain the optimal balance, resulting in a lower reduction rate and reconstruction accuracy. If the fixed threshold is smaller than expected, less data are reduced and data reconstruction becomes easier. While the reconstruction accuracy is superior, the data reduction rate is compromised, leading to unacceptable energy and bandwidth consumption. On the other hand, if the fixed threshold is excessive, too much data are reduced, and it is difficult to reconstruct accurate data at a remote processor. Experiments in the literature [6] also showed that as the reduction parameter increased, the degree of simplification of a reduced object also increased. Thus, we consider dynamically adjusting the threshold to increase the reduction effect in a dynamic IoT. When IoT data change frequently, the threshold can be lower to upload additional data for reconstruction. As the data stabilize, the threshold can gradually increase to reduce more unessential data.

Aiming to dynamically balance the reduction rate with the reconstruction accuracy, an autonomous IoT data reduction method based on an adaptive threshold is proposed. The proposed method consists of a data reduction phase and a data reconstruction phase. During the reduction phase, concept drift detection is performed to capture IoT data changes and trigger threshold adjustment. The threshold is adjusted to be lower if concept drift occurs, and higher otherwise. During reconstruction, data trends are introduced to improve reconstruction accuracy. When concept drift detection identifies data changes, a data trend is introduced to replace a fixed linear rate from a Kalman filter for higher data reconstruction accuracy. To verify the applicability of the proposed method, experiments are executed in seven properties on three datasets, including stationary and nonstationary types. Then, a comparative analysis with the basic Kalman filtering algorithm [7], LMS filter algorithm [8], and critical+PIP algorithm [9] is conducted. Moreover, our main contribution is as follows.

- First, to the best of our knowledge, this is the first scheme to incorporate an adaptive reduction threshold into a data reduction algorithm based on Kalman filtering, which enables autonomous IoT data reduction without the need for cloud.
- Second, aiming to execute reduction threshold adjustment dynamically, a concept drift detection to capture IoT changes is introduced.
- Third, we add a data trend in the data reconstruction stage to further improve data reconstruction accuracy.

The rest of the paper is organized as follows. In Section 2, we analyze the lack of autonomy from traditional data reduction and several IoT data reduction algorithms, then give essential background knowledge of Kalman filtering and data reduction based on prediction. The autonomous IoT data reduction algorithm is presented in Section 3 in the order of two steps. Section 4 consists of experimental evaluations of data reduction rate and data reconstruction accuracy on stationary and nonstationary datasets. Finally, Section 5 concludes the paper and provides insights on autonomous edge data reduction.

## 2. Related Works

In related works, first, they introduce data compression and data aggregation methods in wireless sensor networks and why they are less autonomous and suitable for a dynamic IoT. Second, data reduction methods in IoT are introduced, especially data prediction that takes into account a dynamic IoT. After that, we summarize the basic process and mathematical basis of using a Kalman filter for data reduction. This process and mathematical notation will continue to be used for the rest of this article.

### 2.1. Data Compression

Data compression techniques [10,11], also known as compressive sampling or compressive sensing, are based on the inherent sparsity properties of natural signals and reduce the original signal according to the Shannon–Nyquist theorem. Data compression can significantly reduce the energy consumption used for data acquisition in IoT nodes. For example, Chang et al. [12] applied the mean difference (MD) for filtering data noise and redundant values in the proposed an AIoT architecture. Gilles et al. [13] used a compressed sensing approach based on a sub-Nyquist scheme, known as a modulated wideband converter, to solve wideband spectrum sensing. Aniol et al. [14] proposed an algorithm based on linear prediction that can perform both the lossless and near-lossless compression of RF signals. The proposed algorithm is coupled with two signal detection methods to determine the presence of relevant signals and apply varying loss levels as needed. In data compression, the amount of reduced data depends on the compression algorithm, and thus, the reduction rate can rarely be adjusted autonomously according to the dynamic IoT. Meanwhile, the real-time compression and decompression also put pressure on the storage and computing capabilities of IoT devices.

### 2.2. Data Aggregation

Data aggregation [15] is mainly used at the sink node to regulate sensor sampling frequency and thus optimize energy consumption. It works in two ways. First, it dynamically adjusts the sensor-sampling-frequency-based variance between sensor data at a given epoch, which reduces the energy consumption of the sensing unit by preventing the sensor from collecting redundant information. Second, it dynamically adjusts the rate at which features are computed from the original signal. Chen et al. [16] proposed to extract the data features based on fast Fourier transform (FFT) and apply K-means to generate a set of patterns to represent the time-series data in the application of reducing real-time bridge vibration data. Wang et al.  [17] proposed an energy-efficient load balancing tree-based data aggregation scheme (LB-TBDAS) for grid-based WSNs. In the scheme, the sensing area is partitioned into many cells of a grid, and the treelike path is established by using the minimum spanning tree algorithm. Zhang et al. [18] proposed a lightweight and privacy-friendly data aggregation scheme against abnormal data, in which the valid data can correctly be aggregated, but abnormal data will be filtered out during the aggregation process. Data aggregation emphasizes the task allocation of data reduction and reconstruction at the physical level. Then, additional data reduction algorithms are required at each node. In this paper, the sensor performs data reduction, and the remote processor performs reconstruction.

### 2.3. IoT Data Reduction

To reduce data transferred to an edge node, Bhargava et al. [19] came up with the idea of only storing values that cannot be predicted accurately based on history. According to an analysis of geographical restrictions from Cao et al. [20], only data along the trajectory for local services should be collected. Wang et al. [21] built an RNN by edge-cloud cooperating for performing data prediction on the edge node and selecting necessary data for updating the data prediction model to upload. The edge data were divided into known situations and unknown scenarios by Zhang et al. [22] for learning model updates. Only the recognized unknown situations are sent to the cloud, and other redundant data are discarded.

An in-networking approach is proposed in [7] based on data prediction. The proposed approach consists of data filtering and data fusion layers. The data filtering layer aims to minimize the number of transmissions. At the same time, the data fusion layer fuses the data based on the minimum squared error criterion. Its Kalman filter double-layer architecture is used in this paper as the base model and comparison method. The least mean square (LMS) algorithm is proposed in [8]. The algorithm is based on two decoupled LMS windowed filters combined convexly with different sizes. It estimates future readings at both the sink and sensor nodes. Data transmission occurs if the current reading deviates significantly from a predefined threshold.

Given the dynamic nature of IoT, several existing data prediction approaches focus on dynamic edge resources and sensor hardware for data reduction. Fuzzy redundancy elimination for data deduplication (FREDD) [23] finds that traditional data reduction overlooks the context and dynamics of the network, meanwhile relying on a fixed threshold to execute data reduction. Simple natural language rules represent domain knowledge and expert preferences regarding data duplication boundaries. It is adapted for multiple scenarios, considering both static and mobile devices, with different configurations of hard-separated and soft-separated zones and sensor coverage areas. Data redundancy management for leaf-edges (DRMF) [24] allows for identifying and removing data redundancies in connected environments at the device level. DRMF considers static and mobile edge devices and provides two temporal and spatiotemporal redundancy detection algorithms. Once redundancies are identified, DRMF performs data deduplication, considering the dynamic requirements of data consumers and device resources. Meanwhile, data inaccuracies and unreliability due to sensor dynamics are usually ignored [5]. Thus, data reduction and faulty data detection are proposed while enhancing data reliability.

The following is a summary of how our approach differs from other data reduction techniques. First, existing data reduction techniques rarely consider the dynamic balance between reduction rate and reconstruction accuracy in a dynamic IoT. Second, we adaptively adjusted thresholds for autonomous data reduction using concept drift detection [25]. Finally, current Kalman-filter-based data prediction techniques assume that the IoT data vary linearly [26] due to low computing capabilities of sensors. They do not use an adaptive data trend [27] to forecast future data.

### 2.4. Kalman Filtering Basics

To introduce data reduction based on Kalman filtering, we give a brief review of Kalman filtering [26], which contains two steps, named the prediction step and the correction step. The prediction step can be described as

(1) $$x_{k}=A_{k}x_{k-1}+B_{k}u_{k}$$

(2) $$P_{k}=A_{k}P_{k-1}A_{k}^{T}+Q_{k},$$

where $x_{k}$ is the estimate of the state at time step *k*, $A_{k}$ is the state transition matrix, $B_{k}$ is the control input matrix, $u_{k}$ is the control input, $P_{k}$ is the estimate of the covariance matrix of the state estimate, and $Q_{k}$ is the process noise covariance matrix.

The correction step can be described as

(3) $$y_{k}=z_{k}-H_{k}x_{k}$$

(4) $$S_{k}=H_{k}P_{k}H_{k}^{T}+R_{k}$$

(5) $$K_{k}=P_{k}H_{k}^{T}S_{k}^{-1}$$

(6) $$x_{k}=x_{k}+K_{k}y_{k}$$

(7) $$P_{k}=\left(I-K_{k}H_{k}\right)P_{k},$$

where $y_{k}$ is the innovation, $z_{k}$ is the measurement, $H_{k}$ is the measurement matrix, $S_{k}$ is the covariance of the innovation, $K_{k}$ is the Kalman gain, and $R_{k}$ is the measurement noise covariance matrix.

In basic data prediction based on Kalman filtering methods, $z_{k}$ denotes the real-time data collected by the sensor, and $x_{k}$ the data predicted by the Kalman filter. Data prediction executes by building a data prediction model with the same Kalman filter parameters at both the sensor and the remote processor. The data predicted by the sensor and the remote processor are identical at one time. Therefore, the sensor only needs to determine if the predicted data $x_{k}$ are accurate or not before uploading the data. No data transmission is necessary if the difference between the predicted value $x_{k}$ and the collected value $z_{k}$ is smaller than the reduction threshold.

(8) $$e_{k}=\left|z_{k}-x_{k}\right|$$

When $e_{k}$ calculated by Equation (8) is less than $e_{max}$, the error is accepted, and data do not need to be uploaded. The parameter $e_{max}$ determines the accuracy tolerance and reconstruction accuracy. Thus, the value of $e_{max}$ is crucial in balancing the reduction rate with reconstruction accuracy.

## 3. Proposed Adaptive Reduction Threshold Data Reduction Method

**Figure 1:**
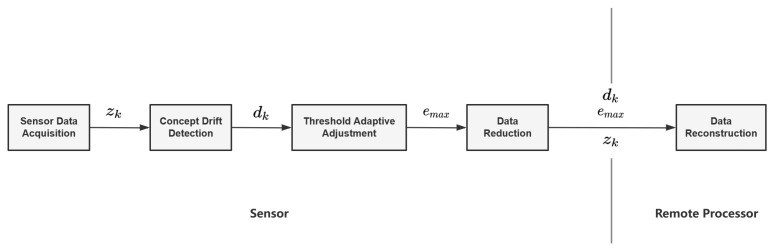
Proposed adaptive reduction threshold data reduction method.

Aiming to dynamically balance the reduction rate with reconstruction accuracy, we propose an autonomous IoT data reduction method based on an adaptive threshold. The proposed method consists of five modules: sensor data acquisition, concept drift detection, threshold adaptive adjustment, data reduction, and data reconstruction. As shown in Figure 1, the modules are divided into two main components: the sensor and the remote processor. The sensor is responsible for data acquisition and reduction, while the remote processor is for data reconstruction.

Sensor data are transmitted to the concept drift detection after a sensor data acquisition module. The concept drift detection module is responsible for detecting IoT data changes. If concept drift is found, the adaptive threshold adjustment module lowers the threshold $e_{max}$. In other cases, $e_{max}$ rises and transmits to the data reduction module. The basic Kalman filter was used to execute the reduction in the data reduction module. Next, it was chosen whether to transmit the real data $z_{k}$ to the remote processor based on the comparison with the threshold. If $x_{k}$ is similar to $z_{k}$, there is no need to transmit $z_{k}$ to a remote processor, and a remote processor uses $x_{k}$ predicted locally in the same parameters with a sensor. Otherwise, $z_{k}$ should transmit to a remote processor and be assigned to $z_{k}$ for accurate later prediction. This assignment is an update to the remote processor’s Kalman filter, which failed to predict at time k and needs to be updated for later predictions. Without $z_{k}$, the data reconstruction module forecasts $x_{k}$ based on the data trend $d_{k}$ and Kalman filter. Each algorithm is analyzed in subsections next in this paper.

### 3.1. Adaptive Adjustment for Reduction Threshold Based on Concept Drift Detection

As mentioned above, adaptive threshold adjustment based on concept drift detection is vital for balance reduction rate and reconstruction accuracy. The detected concept drift indicates a change in the data pattern in a given time window, necessitating a lower data reduction rate to capture more data. Without drift, the data reduction rate gradually increases to filter out irrelevant data.

The Kalman filter assumes that observed data vary linearly [26] and that the linear change rate is constant. Since IoT is dynamic, the linear rate may change sometimes. The linear rate will likely change when the absolute value of a cumulative increment over a time window is abnormal. Thus, the cumulative sum (CUSUM) algorithm [28] is employed to detect concept drift. CUSUM is a statistical control method that detects small shifts in the mean value of a process by monitoring it over time. The CUSUM algorithm accumulates and amplifies persistent biases, thus allowing earlier detection of concept drift, such as linear rate changes. Furthermore, we demonstrate that the CUSUM algorithm can be integrated with other concept drift detection methods by merely swapping out the drift detection module with a different algorithm.

The algorithm works as follows. To address detected concept drift, if the current value of $e_{max}$ exceeds the established $error_{min}$, $e_{max}$ decreases to lower the reduction rate and enhance the reconstruction accuracy. Without concept drift, $e_{max}$ increases for a higher reduction rate. Adjustments of $e_{max}$ are subject to the constraint that they must remain within the specified $error_{max}$ and $error_{min}$. When the values of $error_{max}$ or $error_{min}$ are large, a higher data reduction rate is chosen at the expense of a lesser level of reconstruction accuracy, which is suited for sensors with limited processing power. When $error_{max}$ or $error_{min}$ is small, a higher reconstruction accuracy can be guaranteed instead of pursuing a higher data reduction rate. More complex and intelligent decision-making behaviors can be performed based on more accurate data. The settings of $error_{max}$ and $error_{min}$ need to be analyzed and set after particular experiments on different datasets. In the experimental section of this paper, the data reduction rate and reconstruction accuracy are compared and analyzed in detail for different threshold values. Meanwhile, the step size of each threshold change depends on the experimental setup and preferences for how fast or slow the concept drift needs to be adapted.

### 3.2. Autonomous Data Reduction Algorithm Based on Adaptive Reduction Threshold

Next, we describe how to execute autonomous data reduction with an adaptive threshold. In addition, a mechanism for calculating and uploading data trend is shown. When the data are initialized, $z_{1}$ is uploaded and stored into the cachedval. With using historical data, cache the actual value before calculating $d_{k}$. To determine whether concept drift has taken place and to establish the new threshold, Algorithm 1 is performed. If the threshold value has changed, it suggests there may have been a change in the linear rate, in which case, $d_{k}$ should be uploaded instead of $H_{k}$ to forecast future data. Uploading $d_{k}$ is not necessary in any other case. The estimated value $x_{k}$ is then calculated using the Kalman filter, and the gap between the estimated value and the actual value is compared with $e_{max}$. The real value $z_{k}$ should be submitted when the difference exceeds $e_{max}$.

(9) $$d_{k}=\left\{\begin{array}{cc} z_{k}-z_{k-1}, & k=2\\ \alpha \left(z_{k}-z_{k-1}\right)+\left(1-\alpha \right)d_{k-1}, & k>2 \end{array}\right.$$

**Algorithm 1** Adaptive Adjustment Algorithm for Reduction Threshold
**Input:** current threshold $e_{max}$, threshold minimum $error_{min}$, threshold maxmum $error_{max}$
  1:**while** True **do**  2:    Call the CUSUM algorithm to determine if concept drift has occurred  3:    **if** Concept drift occurs **then**  4:        **if** $e_{max}$ > $error_{min}$ **then**  5:           Lower the current threshold $e_{max}$  6:           **if** $e_{max}$ < $error_{min}$ **then**  7:               $e_{max}$ = $error_{min}$  8:    **else**  9:        **if** c < $error_{max}$ **then**10:           Raise the current threshold $e_{max}$11:           **if** $e_{max}$>$error_{max}$ **then**12:               $e_{max}$ = $error_{max}$


In Equation (9), $d_{k}$ represents the data trend at k and is smoothed with a weight α, which lies in the range [0, 1]. A value of α close to 1 prioritizes the most recent trend. Both the true data value and data trend $d_{k}$ are transmitted to the remote processor for data reconstruction, as shown in Algorithm 2.
**Algorithm 2** Data Reduction Algorithm Based on Adaptive Reduction Threshold**Input:** current threshold $e_{max}$, sensor reading $z_{k}$, data trends $d_{k-1}$, data cache cachedval  1:**while** True **do**  2:    **if** k = 1 **then**  3:        Insert $z_{k}$ into cachedval  4:        Send $z_{k}$ to remote processor  5:    **else**  6:        Insert $z_{k}$ into cachedval  7:        Calculate $d_{k}$  8:        Call Algorithm 1 to calculate adaptive reduction threshold  9:        **if** $e_{max}$ changes **then**10:           send $d_{k}$ to edge server11:        Call the Kalman filter to calculate estimated value $x_{k}$12:        $e_{k}$ = $z_{k}$ − $x_{k}$13:        **if** |$e_{k}$| > $e_{max}$ **then**14:           send $z_{k}$ to remote processor

Concept drift indicates the possibility of linear rate change, invalidating the original Kalman filter assumption of a constant linear rate. As a result, $d_{k}$ should be submitted for prediction instead of $H_{k}$. The observation does not match the sensor’s predicted value when $e_{k}$ is larger than the threshold, and it is also challenging to reconstruct. However, these may be anomaly data or a measurement error rather than a concept drift or linear rate change. In this scenario, $H_{k}$ remains valid to forecast future data.

### 3.3. Data Reconstruction Algorithm Based on Data Trend

After autonomous data reduction, the remote processor does not receive the data uploaded from the sensor in every time window. When the data processor receives $z_{k}$, there is no need for data reconstruction. Nevertheless, when the remote processor fails to receive sensor data, data reconstruction is performed using a Kalman filter assisted by the data trend $d_{k}$. The data reconstruction procedure is detailed in Algorithm 3. The Kalman filter assumes that the observed data vary linearly. Since IoT is dynamic, nonlinear changes could occur sometimes. Nonlinear Kalman filters, however, are challenging to implement in IoT due to limited computing and storage capacity. As a result, when concept drift detection identifies data changes, we use a data trend to replace the fixed Hk and forecast future value.
**Algorithm 3** Data Reconstruction Algorithm Based on Kalman Filtering and Data Trend**Input:** sensor reading $z_{k}$, data trends $d_{k}$, data cache cachedval  1:**while** True **do**  2:    **if** sensor reading $z_{k}$ is not None **then**  3:        Insert $z_{k}$ into cachedval  4:        Calculate $d_{k}$  5:    **else**  6:        Call the Kalman filter to calculate estimated value $x_{k}$  7:        **if** |$e_{k}$| > $e_{max}$ **then**  8:           $z_{k}$ = $z_{k-1}$ + $d_{k}$  9:        **else**10:           $z_{k}$ = $x_{k}$

Upon receiving of the data $z_{k}$ from the sensor, the Kalman filter at the remote processor undergoes a data reconstruction phase. Data trends are stored to facilitate data reconstruction in subsequent cycles. Reversely, the Kalman filter is utilized to predict $z_{k}$ based on $x_{k}$. First, the difference between the Kalman filter’s predicted value $x_{k}$ and the reconstructed data is calculated. If this difference exceeds a specified threshold $e_{max}$, the trend of the data $d_{k}$ is utilized for reconstruction. Otherwise, the result of the Kalman filter is employed as the reconstruction outcome.

## 4. Experiments

### 4.1. Datasets and Experiment Setting

For the experiments, three datasets were selected for analysis. The first dataset, Intel Lab data (Bodik P, Hong W, Guestrin C. Intel Lab data. http://db.csail.mit.edu/labdata/labdata.html, 2004), comprises information on data collected from 54 sensors deployed at Intel Lab from 28 February 2004, to 5 April 2004. Data were collected at a frequency of 30 seconds per sample, and temperature, humidity, light, and voltage properties were included. Experimental comparisons were performed using 6000 temperature, humidity, and light sensor data from this dataset. The second dataset, the Individual Household Electric Power Consumption dataset (Lichman M, UCI Machine Learning Repository. University of California, Irvine, School of Information and Computer Sciences, 2013), encompasses 2,075,259 measurements collected from December 2006, to November 2010 in residences in Sceaux, France. The data were acquired at 60 seconds per sample frequency and included attributes such as voltage, current, and power. Experimental comparisons were performed using 6000 voltage, current, and power sensor data from this dataset. The third dataset is the Dodgers Loop Sensor dataset (Lichman M, UCI Machine Learning Repository. University of California, Irvine, School of Information and Computer Sciences, 2013), which contains data collected from 10 April 2005, to 1 October 2005, on the Glendale ramp of the Los Angeles 101 North Freeway. Experimental comparisons were performed using 6000 data points within this dataset.

Upon conducting ADF root mean square tests on the above properties, we found *p*-values of 0.937 and 0.9024 for Intel Lab data, and 0.7437 and 0.7598 for current and power in the Household Power Consumption data. These values were significantly higher than 0.05, leading to the acceptance of the null hypothesis $H_{0}$ and indicating that the data exhibited stationary patterns. In contrast, ADF test results for the illumination attribute in Intel Lab data, voltage attribute in Household Power consumption data, and vehicle count attribute in Dodge Loop Sensor Data reveal *p*-values of $6.54\times 10^{-16}$, $1.31\times 10^{-12}$ and 0.0, respectively. These values were close to 0, leading to the rejection of $H_{0}$ and suggesting that these data exhibited nonstationary patterns, as shown in Table 1.

This paper compared two aspects to evaluate the effectiveness of the proposed method in data reduction: data reduction rate (DRR) and data reconstruction accuracy (DRA). The definition of the data reduction rate is shown in Equation (10), where DRR represents the data rate, AD represents the total amount of data, and RD represents the total amount of remaining data after reduction. The data reconstruction accuracy is inspired by the Jaccard similarity between reconstructed and original data of the same length. Let $T_{1}=\left[z_{1},z_{2},\cdots ,z_{n}\right]$ be the actual collected data, and $T_{2}=\left[r_{1},r_{2},\cdots ,r_{n}\right]$ be the reconstructed data. The Jaccard similarity between $T_{1}$ and $T_{2}$ is calculated using Equation (11), where DRA represents the data reconstruction accuracy, and n represents the number of reconstructed data. Meanwhile, we calculate the transmission of $d_{k}$ when computing the transmission of our method.

(10) $$DRR=\frac{AD-RD}{AD\times 100}$$

(11) $$DRA=\frac{\sum_{i=1}^{n}\min\left(z_{i},r_{i}\right)}{\sum_{i=1}^{n}\max\left(z_{i},r_{i}\right)\times 100}$$

### 4.2. Experiments on Adaptive Reduction Threshold $e_{max}$

To ensure that the threshold varies within a specific range, the algorithm is executed on the same dataset, the range of threshold variation is calculated and shown in Table 2 and Table 3. The calculated range of threshold variation is also used as a statistical value in subsequent data reduction comparison experiments.

Experiments were conducted using the proposed data reduction method on the temperature and humidity attributes of the Intel Lab data dataset, as well as the current and power characteristics of the Household Power Consumption dataset with stationary-type variations. The threshold range for the temperature attribute was set between 0.01 and 0.1 °C, with an average adaptive threshold of 0.0598 °C, a median threshold of 0.07 °C, a mode threshold of 0.09 °C, and a Pearson correlation coefficient of −0.432 between the threshold variation process and the temperature attribute. The threshold range for the humidity attribute was set to 0.01–0.14%, with an average adaptive threshold of 0.0654%, a median threshold of 0.06%, a mode threshold of 0.01%, and a Pearson correlation coefficient of 0.4882 between the threshold variation process and the humidity attribute. For the current attribute of the Household Power Consumption dataset, the threshold range was set between 0.2 A and 4 A, with an average adaptive threshold of 2.45 A, a median threshold of 3.4 A, a mode threshold of 4 A, and a Pearson correlation coefficient of −0.548 between the threshold variation process and the current attribute. The threshold range for the power attribute was set between 0.25 and 7.2 W, with an average adaptive threshold of 2.26 W, a median threshold of 0.85 W, a mode threshold of 0.25 W, and a Pearson correlation coefficient of 0.5003 between the threshold variation process and the humidity attribute. The threshold variation for all attributes showed a moderate correlation with the Data, demonstrating the effectiveness of the proposed dynamic threshold adjustment mechanism in the Data reduction mechanism. The adaptive adjustment mechanism based on concept drift detection can adjust the reduction rate as the data change pattern evolves.

**Figure 2:**
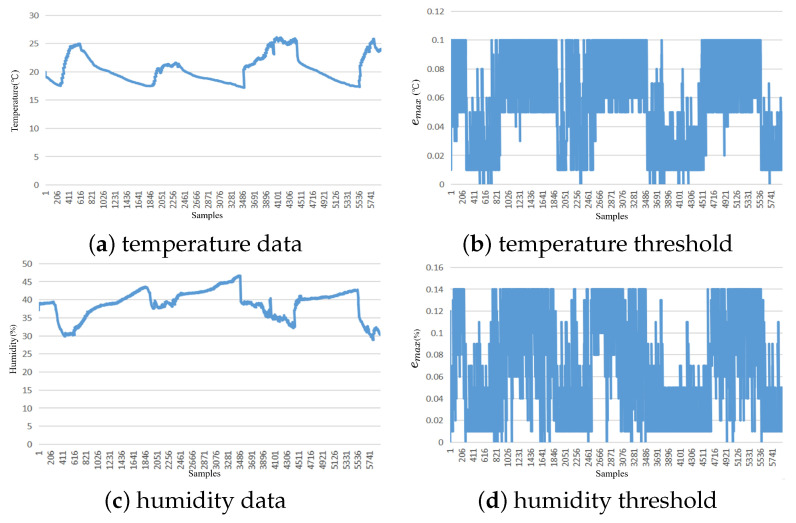
Adaptive threshold chart of Intel Lab data.

**Figure 3:**
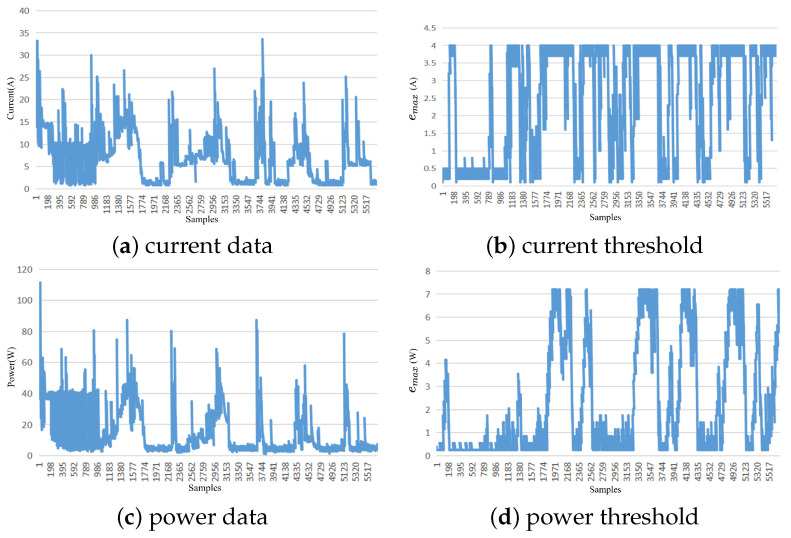
Adaptive threshold chart of Household Power Consumption.

The following two figures depict the threshold variation process of stationary data. Figure 2 corresponds to the Intel Lab dataset, where Figure 2a shows the temperature data change, Figure 2b shows the temperature threshold change, Figure 2c shows the humidity data change, and Figure 2d shows the humidity threshold change. Figure 3 corresponds to the Household Power Consumption dataset, where Figure 3a shows the current data change, Figure 3b shows the current threshold change, Figure 3c shows the power data change, and Figure 3d shows the power threshold change.

In this study, the proposed method for data reduction was applied to nonstationary data from the Intel Lab data for light intensity, the Household Power Consumption dataset for voltage, and the Dodgers Loop Sensor dataset for vehicle count. The threshold range for the light intensity attribute was set from 0.1 to 0.9 Lux, with an adaptive threshold average of 0.5139 Lux, a median threshold of 0.5 Lux, and a mode threshold of 0.4 Lux. The threshold change process showed a weak negative correlation with the current attribute, with a Pearson correlation coefficient of −0.398. For the voltage attribute, the threshold range was set from 0.21 to 1.5 V, with an adaptive threshold average of 1.02 V, a median threshold of 1 V, and a mode threshold of 1 V. The threshold change process showed a weak positive correlation with the humidity attribute, with a Pearson correlation coefficient of 0.344. For the Dodgers Loop Sensor, the vehicle count threshold range was set from 1 to 7, with an adaptive threshold average of 1.383, a median threshold of 1, and a mode threshold of 1. The threshold change process showed a weak negative correlation with the humidity attribute, with a Pearson correlation coefficient of –0.372. When dealing with nonstationary data, the threshold change process is weakly correlated with the data attributes. The data fluctuation is relatively large, resulting in significant differences between adjacent data points. Consequently, the Kalman filter model may fail to predict the next data value accurately, and the error threshold will continue to decrease. The error threshold is maintained at a relatively low level to ensure data accuracy while the data reduction rate is decreased.

**Figure 4:**
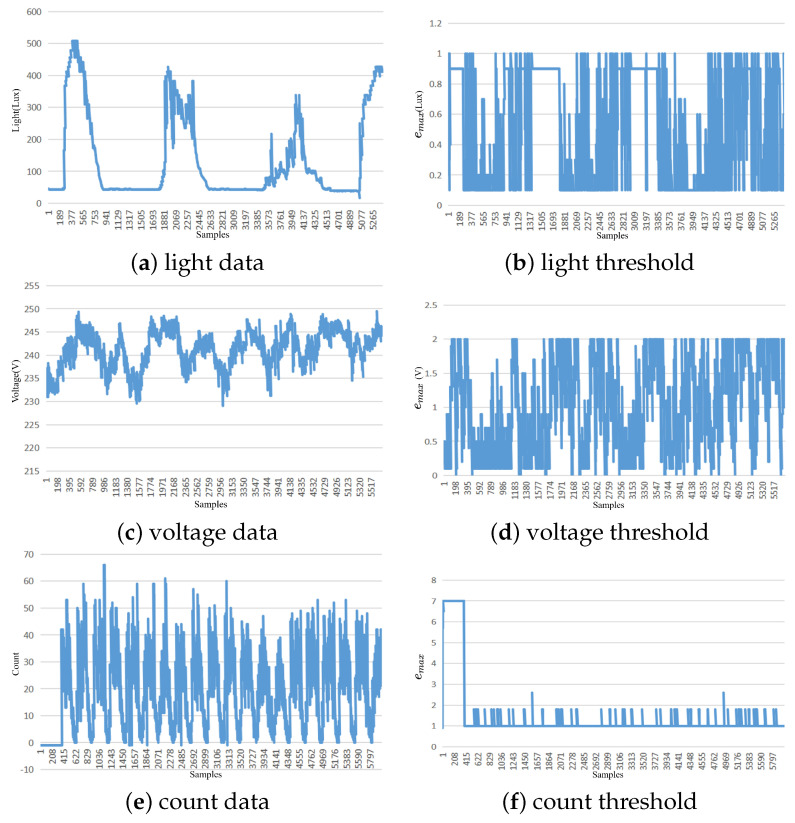
Adaptive threshold chart of nonstationary datasets.

The following images depict the threshold variation process for nonstationary data. Figure 4a shows the change in light data, Figure 4b represents the corresponding threshold variation, Figure 4c shows the variation in voltage intensity, and Figure 4d shows the voltage threshold variation. Figure 4e illustrates the variation in count data, while Figure 4f presents the corresponding threshold variation.

After $error_{max}$ and $error_{min}$ are set, we calculate a tenth of the difference between $error_{max}$ and $error_{min}$ as the step size. Each time $e_{max}$ increases or decreases, it changes by one step.

### 4.3. Experiments on Adaptive Reduction Rate and Reconstruction Accuracy

In this section, the effectiveness of the proposed method is validated for both stationary and nonstationary datasets by comparing it with fixed threshold reduction methods, such as basic Kalman filter and LMS filter reduction methods, as well as the non-threshold reduction method and the critical+PIP reduction method. The proposed method adjusts the reduction rate dynamically based on the data change pattern, and the reduction threshold changes during the reduction process. Due to various external factors that affect the data, the change patterns of single-dimensional sensor data may differ at different stages, leading to differences in data reduction rate and reconstruction accuracy. Therefore, the minimum, maximum, mean, and mode of the threshold values that are adaptively adjusted by the proposed method in different datasets are taken as the fixed threshold values in traditional methods for comparison with basic Kalman filter and LMS filter data reduction methods. The critical+PIP algorithm is a non-threshold data reduction algorithm, and its efficiency is measured by comparing the data reduction rate and data reconstruction accuracy of the critical+PIP algorithm under both stationary and nonstationary datasets.

#### 4.3.1. Experiments on Stationary Attributes Compared with Fixed Threshold

By conducting experiments on temperature and humidity data from Intel Lab data, it is found that the proposed method has a higher data reduction rate than the basic Kalman and LMS filter data reduction methods with threshold values set by mean, median, and mode. As shown in Table 4 and Table 5, when the threshold is set as the maximum value, the proposed method has only a slight reduction rate lower than that of Kalman and LMS filter. Moreover, the data reconstruction accuracy of the proposed method is higher than that of traditional Kalman and LMS filter data reduction methods with threshold values set by mean and median.

Through a comparative experiment on the current and power data in the Household Power Consumption dataset, it is found that for stationary datasets, the data reduction rate of the proposed method is higher than that of the basic Kalman filter with mean or median as the threshold. As shown in Table 6 and Table 7, the data reconstruction accuracy is better than that of basic Kalman filtering and LMS filtering under different threshold values.

For stationary datasets, as the reduction threshold increases, the data reduction rate of the basic Kalman filter and LMS filter will continue to increase, but the data reconstruction accuracy will decrease. The data reduction rate and reconstruction accuracy of the traditional Kalman filter are both higher than those of the LMS filter. Compared with the proposed method, the data reduction algorithm is a dynamic mechanism for controlling the reduction rate, which can adjust the reduction rate dynamically according to the changing patterns of the data. The proposed method achieves higher data reconstruction accuracy when the data reduction rate is equal to that of the traditional Kalman filter.

#### 4.3.2. Experiments on Stationary Attributes Compared with Critical+PIP

The critical+PIP algorithm is a non-threshold-based data reduction algorithm. This article measures the efficiency of the proposed algorithm by comparing its data reduction rate and data reconstruction accuracy with those of another algorithm in the Intel Lab data dataset, specifically for the current and power attributes of Household Power Consumption and the temperature and humidity attributes.

The experimental results in Table 8 show that the data reconstruction accuracy of the critical+PIP algorithm is unstable. To observe the difference in data reconstruction accuracy between the two algorithms, the data reduction rate is controlled between 20% and 80%. For the Intel Lab data, the temperature data reconstruction accuracy of the critical+PIP algorithm decreased from 91.28% to 69.73%, and the humidity data reconstruction accuracy decreased from 68.91% to 52.60%. When processing current data, the data reconstruction accuracy of the critical+PIP algorithm decreased from 99.12% to 73.97%, and when processing power data, the data reconstruction accuracy decreased from 97.10% to 60.49%. The proposed method achieves higher and more stable data reconstruction accuracy with the same data reduction rate.

#### 4.3.3. Experiments on Nonstationary Attributes Compared with Fixed Threshold

In the following, we will compare the fixed threshold methods in the nonstationary attributes. Analysis of the Intel Lab light data indicates that the data values remain mostly unchanged most of the time. As shown in Table 9, the proposed method exhibits a similar data reduction rate and data reconstruction accuracy to those of the other compared methods. We calculate the transmission of $d_{k}$ when computing the transmission of our method. Therefore, the effect may not be significant in light attribute in Intel Lab data, which may be caused by the fact that there are fewer nonlinear cases and $d_{k}$ does not need to be transmitted.

In the case of voltage data shown in Table 10 and count data shown in Table 11, the proposed method achieves a data reduction rate that is 10% lower than that of the traditional Kalman filter with a mean value threshold for nonstationary datasets. However, the data reconstruction accuracy of the proposed method is better than that of the traditional Kalman filter and LMS filter under different threshold conditions.

When dealing with nonstationary datasets, the data reduction rate of the traditional Kalman filter and LMS filter will continue to increase with the increase in the reduction threshold. Still, the data reconstruction accuracy will be very low, and sudden changes in data anomalies cannot be observed in a timely manner. In the case of processing nonstationary datasets, the proposed method can automatically reduce the data reduction rate, maintain sensitivity, and sustain high data accuracy.

#### 4.3.4. Experiments on Nonstationary Attributes Compared with Critical+PIP

This subsection will measure the efficiency of the proposed algorithm by comparing the data reconstruction accuracy of critical+PIP data reduction methods with the same data reduction rate. As Shown in the Table 12, the data reconstruction accuracy of the critical+PIP algorithm is not stable. When dealing with light data, the data reduction rate is controlled from 50% to 80%, the data reconstruction accuracy of critical+PIP decreases from 88.48% to 77.70%, and the data reconstruction accuracy of the proposed method decreases from 96.69% to 93.05%. When processing voltage data, the data reduction rate is controlled to 20–80%, and the data reconstruction accuracy of critical+PIP data decreases from 92.36% to 61.73%, while the data reconstruction accuracy of the proposed method in this paper decreases from 97.35% to 80.36%. It can be seen that the proposed method in this paper has higher data reconstruction accuracy with the same data reduction rate, while the data reconstruction accuracy is more stable, and the data reduction effect is better. The data reconstruction accuracy of the critical+PIP algorithm decreases from 75% to 64.54%, and the data reconstruction accuracy of this paper decreases from 83.24% to 67.70%. This paper’s data reconstruction accuracy is higher with the same data reduction rate. Meanwhile, the data reconstruction accuracy of this paper’s method is more stable than that of the critical+PIP algorithm when facing a nonstationary dataset.

## 5. Conclusions

The large amount of data generated by the sensor needs to be reduced at the sensor and subsequently reconstructed to save bandwidth and energy. As the reduced data increase, the reconstructed data become less accurate. The trade-off between reduction rate and reconstruction accuracy is commonly controlled by the reduction threshold, which is calculated by experiments based on historical data. The motivation is that the basic assumption of the Kalman filter is to remove the influence of noise in the case of static linear rate, while a dynamic IoT may have special cases, such as linear rate change and concept drift. Using the original threshold significantly harms the reduction rate and reconstruction accuracy, and persists for long periods of time when concept drift occurs. In order to dynamically balance the reduction rate with the reconstruction accuracy, we propose an autonomous IoT data reduction method based on an adaptive threshold. During the data reduction phase, concept drift detection is performed to capture the IoT dynamic changes and trigger threshold adjustment. During the data reconstruction phase, a trend is added to the data to improve the reconstruction accuracy. The effectiveness of the proposed method is demonstrated by comparing the proposed method with the basic Kalman filtering algorithm, LMS algorithm, and PIP algorithm on stationary and nonstationary datasets. Compared with not applying the adaptive threshold, on average, we have an 11.7% improvement in accuracy for the same reduction rate or a 17.3% improvement in reduction rate for the same accuracy. The proposed approach focuses on addressing ongoing changes autonomously without cloud involvement, rather than short-term fluctuations, such as noise. Not limited to the IoT environment, the autonomous data reduction is also important to enable green and efficient data mining through energy and bandwidth saving.

## Figures and Tables

**Table 1:** Datasets.

Dataset	Attribute	*p*-Value	Decision	Stationary
Intel Lab Data	Temperature	0.937	Retain $H_{0}$	Stationary
	Humidity	0.9024	Retain $H_{0}$	Stationary
	Light	$6.54\times 10^{-16}$	Reject $H_{0}$	Nonstationary
Power Consumption	Voltage	$1.31\times 10^{-12}$	Reject $H_{0}$	Nonstationary
	Current	0.7437	Retain $H_{0}$	Stationary
	Power	0.7598	Retain $H_{0}$	Stationary
Dodgers Loop Sensor	Count	0	Reject $H_{0}$	Nonstationary

**Table 2:** Threshold setting for stationary datasets.

	Intel Lab Data		Household Power Consumption	
Threshold	Temperature	Humidity	Current	Power
Min(e_max)	0.01	0.013	0.21	0.2
Max(e_max)	0.09	0.14	1.5	4

**Table 3:** Threshold setting for nonstationary datasets.

	Intel Lab Data	Household Power Consumption	Dodgers Loop Sensor
Threshold	Light	Voltage	Count
Min(e_max)	0.1	0.21	1
Max(e_max)	0.9	1.5	7

**Table 4:** Experimental results of temperature data.

	Basic Kalman Filter		LMS Filter		Proposed Method	
	DRR	DRA	DRR	DRA	DRR	DRA
Mean (0.07)	64.60%	77.12%	27.10%	98.11%	68.60%	94.11%
Medium (0.05)	59.80%	82.77%	26.30%	99.29%		
Mode (0.04)	56.40%	85.11%	26.00%	99.57%		
Min (0.01)	36.10%	92.61%	25.10%	99.97%		
Max (0.1)	68.10%	69.41%	27.40%	96.35%		

**Table 5:** Experimental results of humidity data.

	Basic Kalman Filter		LMS Filter		Proposed Method	
	DRR	DRA	DRR	DRA	DRR	DRA
Mean (0.06)	60.25%	92.02%	56.60%	59.07%	68.90%	95.42%
Medium (0.06)	60.25%	92.10%	56.60%	63.66%		
Mode (0.01)	32.30%	99.60%	31.25%	99.79%		
Min (0.01)	32.30%	99.60%	31.25%	99.79%		
Max (0.14)	81.85%	67.84%	79.50%	52.06%		

**Table 6:** Experimental resultsof current data.

	Basic Kalman Filter		LMS Filter		Proposed Method	
	DRR	DRA	DRR	DRA	DRR	DRA
Mean (2.45)	69.10%	78.87%	66.65%	75.24%	69.93%	80.81%
Medium (3.4)	93.90%	63.23%	75.70%	63.95%		
Mode (4)	95.50%	61.57%	80.15%	57.42%		
Min (0.2)	58.10%	99.60%	46.05%	98.53%		
Max (4)	95.50%	61.57%	80.15%	57.42%		

**Table 7:** Experimental results of power data.

	Basic Kalman Filter		LMS Filter		Proposed Method	
	DRR	DRA	DRR	DRA	DRR	DRA
Mean (0.85)	53.85%	70.30%	16.60%	72.30%	69.05%	75.57%
Medium (2.26)	67.70%	62.10%	36.95%	61.76%		
Mode (0.25)	22.30%	91.60%	8.35%	88.06%		
Min (0.25)	22.30%	91.60%	8.35%	88.06%		
Max (7.2)	81.95%	67.84%	72.55%	52.11%		

**Table 8:** Experimental results of stationary attributes compared with critical+PIP.

	Intel Lab Data				Household Power Consumption			
	Temperature		Humidity		Current		Power	
DRR	C+PIP	Our	C+PIP	Our	C+PIP	Our	C+PIP	Our
20%	91.28%	97.35%	68.91%	86.03%	99.12%	99.18%	97.10%	97.35%
40%	86.84%	96.17%	66.56%	82.28%	95.69%	98.37%	84.45%	95.73%
60%	75.63%	96.26%	59.57%	80.33%	82.64%	94.40%	78.37%	94.48%
80%	69.73%	95.27%	52.60%	74.35%	73.97%	91.72%	60.49%	93.26%

**Table 9:** Experimental results of light data.

	Kalman Filter		LMS Filter		Proposed Method	
	DRR	DRA	DRR	DRA	DRR	DRA
Mean (0.51)	83.15%	93.89%	82.65%	92.68%	82.30%	93.11%
Medium (0.5)	83.30%	93.89%	82.65%	92.68%		
Mode (0.9)	84.45%	93.94%	82.65%	92.68%		
Min (0.1)	82.75%	93.89%	82.65%	92.68%		
Max (1)	85.65%	93.52%	82.65%	92.68%		

**Table 10:** Experimental results of voltage data.

	Kalman Filter		LMS Filter		Proposed Method	
	DRR	DRA	DRR	DRA	DRR	DRA
Mean (1.02)	82.20%	65.48%	70.80%	60.15%	48.05%	99.85%
Medium (1)	82.00%	65.48%	70.70%	60.15%		
Mode (1)	82.00%	65.48%	70.70%	60.15%		
Min (0.21)	23.45%	97.89%	18.10%	99.96%		
Max (1.5)	94.25%	56.92%	86.25%	49.28%		

**Table 11:** Experimental results of count data.

	Kalman Filter		LMS Filter		Proposed Method	
	DRR	DRA	DRR	DRA	DRR	DRA
Mean (2.13)	55.75%	64.25%	44.35%	65.69%	45.55%	81.46%
Medium (1)	41.80%	75.57%	35.45%	70.43%		
Mode (1)	41.80%	75.57%	35.45%	70.43%		
Min (1)	41.80%	75.57%	35.45%	70.43%		
Max (7)	90.30%	47.96%	35.45%	44.98%		

**Table 12:** Experimental results of nonstationary attributes compared with critical+PIP.

Data Reconstruction Accuracy								
Light			Power			Count		
DRR	C+PIP	Our	DRR	C+PIP	Our	DRR	C+PIP	Our
50%	88.48%	96.69%	20%	92.36%	97.35%	15%	75.00%	83.24%
60%	84.24%	94.69%	40%	86.84%	91.44%	20%	71.21%	75.84%
70%	79.23%	93.69%	60%	75.63%	84.97%	25%	67.30%	72.33%
80%	77.70%	93.05%	80%	61.73%	80.36%	30%	64.54%	67.70%

## Data Availability

The datasets can be obtained from http://db.csail.mit.edu/labdata/labdata.html and http://archive.ics.uci.ed accessed on 1 September 2023.
